# Increasing chronic disease preventive care in community mental health services: clinician-generated strategies

**DOI:** 10.1186/s12888-023-05311-9

**Published:** 2023-12-11

**Authors:** Caitlin Fehily, Belinda Jackson, Vibeke Hansen, Tegan Stettaford, Kate Bartlem, Richard Clancy, Jenny Bowman

**Affiliations:** 1 School of Psychological Sciences, College of Engineering, Science and Environment, The University of Newcastle, Callaghan, NSW Australia; 2 Hunter Medical Research Institute, Clinical Research Centre, New Lambton Heights, NSW Australia; 3 School of Medicine and Public Health, The University of Newcastle, Callaghan, NSW Australia; 4 Hunter New England Mental Health, Hunter New England Local Health District, NSW Health, New Lambton, NSW Australia; 5 School of Nursing and Midwifery, College of Health, Medicine & Wellbeing, The University of Newcastle, Callaghan, NSW Australia

**Keywords:** Chronic Disease, Prevention, Mental health, Preventive care, Community mental health service, Implementation

## Abstract

**Background:**

People with a mental health condition experience a high prevalence of chronic disease risk behaviours e.g., tobacco smoking and physical inactivity. Recommended ‘preventive care’ to address these risks is infrequently provided by community mental health services. This study aimed to elucidate, among community mental health managers and clinicians, suggestions for strategies to support provision of preventive care.

**Methods:**

Three qualitative focus groups (*n* = 14 clinicians) were undertaken in one regional community mental health service to gather perspectives of barriers to preventive care provision, deductively coded against the domains of the Theoretical Domains Framework (TDF). Drawing on the learnings from the focus groups, individual interviews (n = 15 managers and clinicians) were conducted in two services to identify suggestions for strategies to increase preventive care. Strategies were inductively coded and mapped into TDF domains.

**Results:**

Barriers were identified across a wide range of TDF domains, most notably *knowledge* and *environmental context and resources.* Nine strategies were identified across three themes: training, resources and systems changes; mapping to all 14 TDF domains.

**Conclusion:**

Future research seeking to increase implementation of preventive care may be guided by these findings. There is need for greater recognition and resourcing of preventive care as a priority and integral component of mental health treatment.

**Supplementary Information:**

The online version contains supplementary material available at 10.1186/s12888-023-05311-9.

## Background

The median of 10 year reduction in life expectancy experienced internationally by people with a mental health condition is largely a result of elevated morbidity and mortality from chronic physical diseases, and is associated with a high prevalence of modifiable health risk behaviours including: tobacco smoking, poor nutrition, harmful alcohol consumption and physical inactivity [1, 2]. Despite policies directing mental health services to provide ‘preventive care’ to address chronic disease risk behaviours [3] and the existence frameworks to guide its provision [4, 5]; preventive care provision is sub-optimal in international [6–8] and Australian [9–12] mental health services. In Australia, this includes community mental health services, the most frequently accessed specialist mental health service [13]. These services provide outpatient care, employing mental health clinicians of varied professional backgrounds, including psychologists, psychiatrists, social workers, peer workers, mental health nurses, and occupational therapists.

Whilst acknowledging the inequitable physical health experienced by people with a mental health condition, mental health clinicians have identified in previous qualitative research several barriers to providing preventive care for physical health (typically broadly defined and encompassing e.g., nutrition, alcohol, cancer screening, metabolic risk, diabetes, and oral health) [14–18]. This includes barriers such as inadequate time and supportive resources, low clinician confidence [15, 17], clinician perceptions of low consumer interest in change [8], and ambiguity regarding roles and responsibilities [16]. Previous research however has typically focused on the perspectives of mental health nurses and on barriers rather than clinician-generated solutions.

The limited qualitative research investigating mental health clinician recommendations for increasing preventive care has considered physical health broadly, reporting suggestions including additional training [15, 19, 20]; improved communication with other providers, such as general practice [14]; and changes to service delivery including increasing staff and resources, or additional specialist roles [14]. To date, no research has explored community mental health clinicians’ ideas for improving preventive care for the key chronic disease risk behaviours: tobacco smoking, poor nutrition, harmful alcohol consumption and physical inactivity.

Theory-based frameworks have been used in qualitative health research to facilitate the capture of multiple perspectives, shed light on health system complexities, and inform improvements in health policy and service delivery [21, 22]. The Theoretical Domains Framework (TDF) is one such framework that has been recommended as a tool to facilitate comprehensive investigation of factors influencing existing behaviours and guiding intervention development and implementation strategies for clinician behaviour change [23, 24]. The TDF covers 14 theoretical domains that incorporate factors at individual as well as broader systems and environmental levels (see Table 1) [23], and has been used in diverse contexts to identify and prioritise barriers and facilitators to implementation [25–27]. The TDF has also been used to guide researchers and practitioners to select intervention strategies to target the identified barriers and facilitators e.g., to increase shared decision-making in maternity care [28] and to improve cardiovascular health among childhood cancer survivors [29]. No research however has employed the TDF to identify factors influencing preventive care provision for chronic disease risk behaviours in community mental health services, nor to identify clinicians’ ideas for strategies to improve such care. Conducting qualitative research with this aim would provide an in-depth understanding of clinician views, in order to better guide initiatives to increase preventive care.

**Table 1:** Overview of the Theoretical Domains Framework

Domain (definition)	Constructs
**1 Knowledge** An awareness of the existence of something	Knowledge (including knowledge of condition/scientific rationale); Procedural knowledge; Knowledge of task environment
**2 Skills** An ability or proficiency acquired through practice	Skills; Skills development; Competence; Ability; Interpersonal skills; Practice; Skill assessment
**3 Social/professional role and identity** A coherent set of behaviours and displayed personal qualities of an individual in a social or work setting	Professional identity; Professional role; Social identity; Professional boundaries; Professional confidence; Group identity; Leadership; Organisational commitment
**4 Beliefs about capabilities** Acceptance of the truth, reality, or validity about an ability, talent, or facility that a person can put to constructive use	Self confidence; Perceived competence; Self-efficacy; Perceived behavioural control; Beliefs; Self-esteem; Empowerment; Professional confidence
**5 Optimism** The confidence that things will happen for the best or that desired goals will be attained	Optimism; Pessimism; Unrealistic optimism; Identity
**6 Beliefs about consequences** Acceptance of the truth, reality, or validity about outcomes of a behaviour in a given situation	Beliefs; Outcome expectancies; Characteristic of outcome expectancies; Anticipated regret; Consequents
**7 Reinforcement** Increasing the probability of a response by arranging a dependent relationship, or contingency, between the response and a given stimulus	Rewards (proximal/distal, valued/not valued, probable/improbable); Incentives; Punishment; Consequents; Reinforcement; Contingencies; Sanctions
**8 Intentions** A conscious decision to perform a behaviour or a resolve to act in a certain way	Stability of intentions; Stages of change model; Transtheoretical model and stages of change
**9 Goals** Mental representations of outcomes or end states that an individual wants to achieve	Goals (distal/proximal); Goal priority; Goal/target setting; Goals (autonomous/controlled); Action planning; Implementation intention
**10 Memory, attention and decision processes** The ability to retain information, focus selectively on aspects of the environment and choose between two or more alternatives	Memory; Attention; Attention control; Decision making; Cognitive overload/tiredness
**11 Environmental context and resources** Any circumstance of a person’s situation or environment that discourages or encourages the development of skills and abilities, independence, social competence, and adaptive behaviour	Environmental stressors; Resources/material resources; Organisational culture/climate; Salient events/critical incidents; Person x environment interaction; Barriers and facilitators
**12 Social influences** Those interpersonal processes that can cause individuals to change their thoughts, feelings or behaviours	Social pressure; norms; Group conformity; Social comparisons; Group norms; Social support; Power; Intergroups conflict; Alienation; Group identity; Modelling
**13 Emotion** A complex reaction pattern, involving experiential, behavioural, and physiological elements	Fear; Anxiety; Affect; Stress; Depression; Positive/negative affect; Burn-out
**14 Behavioural regulation** Anything aimed at managing or changing objectively observed or measured actions	Self-monitoring; Breaking habit; Action planning

*Note.* Adapted from Cane et al.’s (2012) refined 14 domain framework [23]

The primary aim of this study was to qualitatively explore among mental health clinicians working for two government community mental health services in NSW, Australia, their suggestions for strategies to support routine provision of preventive care for chronic disease risk behaviours (smoking, poor nutrition, harmful alcohol use and physical inactivity), utilising the TDF. Qualitative interviews with clinicians and managers to generate strategies were informed by the prior conduct of focus groups, to investigate perceptions of the barriers to providing such care.

## Methods

### Participants and setting

Participants were staff of two community mental health services within one large health district in regional New South Wales, Australia. The health district had a policy mandating the provision of preventive care in line with the ‘AAR’ Framework: Assess current behaviour levels, offer brief Advice to change identified risk behaviours, and Refer consumers to support services for ongoing behaviour change care [3, 4]. Previous research had noted provision to be sub-optimal [9, 30]. The electronic record system used by the health district incorporated a tool designed to support the provision and recording of AAR for SNAP risks.

The two community mental health services provided care to consumers with a range of diagnoses and severities including anxiety disorders, mood disorders, and schizophrenia; and employed a diverse range of mental health clinicians including: mental health nurses, psychologists, psychiatrists, occupational therapists, and social workers. Staff worked in different clinical teams, including the acute team (seeing consumers transitioning from inpatient to community, requiring a high level of support on a short-term basis) and the rehabilitation and community teams (seeing consumers requiring less acute support, on a long-term basis). ‘Service one’ was the largest in the local health district with approximately 75 clinical staff; while ‘service two’ employed 55 staff. At the time of this study, service one had recently participated in a randomised controlled trial conducted by the research team to determine the effectiveness of embedding a dedicated preventive care clinician in a community mental health service (employed between March and September 2017) [30]. ‘Service two’ did not participate in this trial.

The study employed a combination of focus groups and individual interviews aiming to provide a rich understanding of participant perspectives; a methodology used in previous qualitative research [31–33]. The focus groups were conducted within service one (October 2017), with this methodology enabling a broad understanding of barriers to providing preventive care experienced by staff. The learnings from the focus groups then informed the individual interviews that addressed the primary aim of identifying suggestions for specific solutions or strategies to increase preventive care provision. These interviews were conducted with clinicians and mangers of both services (March-April 2018), with the interview methodology enabling a more specific focus and deep exploration into participants’ suggestions for strategies. This methodology was a reflexive decision made by the research team in response to the conversations in the focus groups focussing predominately on barriers; highlighting the need for additional interviews to understand suggestions for strategies to address these.

This research was approved by the Hunter New England (17/08/16/5.14) and registered with the University of Newcastle (H-2020-0113) Human Research Ethics Committees.

### Recruitment

#### Focus groups

All clinical staff of service one were emailed an information letter about the focus groups by their service manager. Staff were also notified of the opportunity to participate during a staff meeting. Staff were able to email or phone the researchers if they wished to take part; with those expressing interest then contacted to arrange a time to attend a focus group.

#### Interviews

After the conduct of the focus groups, individual interviews were conducted with staff of both mental health services. Two recruitment strategies were used. Firstly, random sampling was undertaken, stratified by professional background, to ensure adequate representation of the different professions working in the services: mental health nurses, psychiatrists, psychologists, other allied health professionals, and management positions. Staff that were randomly selected were emailed by the research team inviting them to take part in an interview. If someone declined, another staff member was randomly selected. Secondly, snowball sampling was also used where participants recommended colleagues who may be interested in taking part, and who were then similarly contacted by the research team to seek consent. Staff were eligible regardless of whether they had participated in the preceding focus groups. Due to data being de-identified it is not possible to know how many/which participants may have participated in both a focus group and interview.

### Data collection

Focus groups and interviews were held during work hours, were audio-recorded and transcribed verbatim. Written, informed consent was obtained from all participants before commencement. Participants were asked to complete a brief demographics questionnaire prior to participating (year of birth, gender, professional background, years worked in mental health, years worked in service). Credibility was ensured by the research team having prolonged engagement with the services from which participants were recruited, the experience of the research team, and the tiered approach to data collection (where information gathered in focus groups informed the interviews).

#### Focus groups

Three focus groups of three to seven people were conducted within service one in October 2017 running for an average of 60 min (range: 48–82 min); facilitated by an experienced independent qualitative researcher (VH), with a co-facilitator (CF) who was a content expert. Groups were conducted using a semi-structured topic guide informed by the TDF to facilitate a comprehensive exploration of barriers to preventive care provision (See Additional File 1 for focus group discussion guide).

#### Interviews

Following the focus groups, individual interviews were conducted by VH across both services either in-person (held at the mental health service; n = 4) or by telephone (n = 11), taking an average of 27 min (range: 15–45 min). Service one interviews were held in March-April 2018, and Service two in August-September 2019. A number of procedural steps were adopted prior to and during the conduct of interviews to ensure that the focus would be on generating possible strategies, rather than barrier identification. To encourage participants to begin thinking broadly about different types of barriers and the range of strategies that might be needed to address them, they were emailed brief written information from the research team at least two days prior to the interview that exemplified potential barriers to clinical care in a range of different health care setting/contexts, framed loosely around TDF domains (see Additional File 2). Interviews commenced with a brief discussion of barriers identified in the focus groups and inviting participants to identify others. Participants were then encouraged to generate possible strategies to address barriers (Additional File 3 contains the interview discussion guide). A reflexive approach was taken, where interview questions and prompts were continually adapted by the interviewer based on information obtained in previous interviews.

### Analysis

Data was entered and coded in NVivo 12.6.0. Analysis followed the guide for using the TDF in implementation research; frequently utilised in studies incorporating multiple data collection methods (focus groups, interviews) [34–36].

Firstly, focus group data were analysed to identify barriers utilising a deductive thematic analysis against a TDF coding framework. In the first stage of data emersion and familiarisation, coders confirmed that a deductive thematic analysis [37] utilising the TDF [38] would be appropriate, as the barriers clearly aligned with TDF domains. Two researchers (BJ and CF) independently coded the first focus group to map identified barriers to the most relevant TDF domain; meeting to discuss any discrepancies and develop a draft codebook. The following focus groups were then independently coded (BJ and CF), with coders meeting frequently to review and revise the codebook in an iterative and reflexive process to ensure themes accurately represented meanings across the whole dataset. Emerging findings were discussed with key members of the services (e.g., managers) to ensure credibility. Information regarding participant professional background and team (e.g., acute, rehabilitation and community) could not be identified on an individual basis; however, where the discussions commented on or reflected such differences these were included in the analysis.

The findings from the deductive thematic analysis of barriers informed the subsequent conduct of individual interviews to identify support strategies. An inductive thematic analysis approach was chosen to draw out themes based on participant experience rather than a priori researcher expectations. Two researchers (pairs of BJ, CF and TS; BJ the consistent coder across all) independently coded interview transcripts. Researchers began by independently coding two transcripts and met to reach consensus on a draft codebook. The remaining transcripts were then coded, with the team regularly meeting to continually revise the codebook collaboratively. Where consensus could not be reached, authors JB and VH were consulted. After final themes and strategies were generated, the strategies were mapped collectively by two researchers (BJ and TS) to the 14 TDF domains. In instances where strategies were relevant to more than one domain, they were coded into multiple domains [39].

## Results

### Sample characteristics

Fourteen clinicians participated across the three focus groups and 15 clinicians and managers in interviews. Sample characteristics are presented in Table 2.

**Table 2:** Sample characteristics

Characteristic	Focus groups (*n* = 14)	Interviews (*n* = 15)
**Gender** (n)		
Male	7	2
Female	7	13
**Professional background** (n)		
Nursing Occupational therapy Social work Psychology Psychiatry Peer worker Dietician	9 1 2 1 1 0 0	5 0 4 3 1 1 1
**Age in years**		
Mean	46.4	42.2
Median	46.0	42.0
**Years worked in mental health**		
Mean	14.6	10.8
Median	15.5	8.0
**Years worked at the service**		
Mean	5.3	5.4
Median	2.0	4.0

*Note.* For interviews, n = 8 were from service one and n = 7 from service two

### Barriers to preventive care provision (focus groups)

Barriers were identified across 13 of the 14 TDF domains, summarised in Table 3. Clinicians held differing perspectives regarding their role in providing preventive care. Some, particularly in the acute care team, believing it to be more the responsibility of other health professionals e.g., GPs (*professional role and identity*). Some clinicians did not have clear intentions to routinely provide preventive care (*intentions*), noting it was not a key clinical goal (*goals*) and it was difficult to determine ‘when’ during treatment and recovery it would be most appropriate to provide it (*memory, attention and decision processes*).

**Table 3:** Identified barriers to providing preventive care and representative quotes

TDF domain	Barriers	Representative quotes
**Knowledge**	Limited awareness of preventive care tools available in the electronic records.	*I know with the Better Health* [electronic tool] *wasn’t really instilled that I had to do that.* (Focus Group 3, male participant 3)
	Unsure of specific services consumers can be referred to.	*Who are the services that we can utilise that might be able to help people with?* (Focus Group 3, female participant 1)
	Unsure of procedures for addressing risk behaviours, including use of electronic tools.	*It’s okay, we’ve found this for the client, now what do we do?* (Focus Group 3, male participant 1)
**Skills**	Lack of skills for provision of preventive care for all SNAP risks, particularly advice/next steps.	*I wonder if a lot of people feel like, well, it’s okay for me to ask the clients this, but what if I find things here that they need to look at, then what do we do? I can’t do this… like you’ve described, I can’t do anything about their nutrition… I don’t feel like I can do anything.* (Focus Group 3, male participant 1)
**Social professional role and identity**	Conflicting perceptions role in providing preventive care, differing by clinical team.	*I think it’s really important to remember as well that when people come into see [the acute team] their priority isn’t their weight, their priority is their mental health, and it’s – [rehab clinician] is in the sort of fortunate position in a lot of respects in that the people that come into to see [rehab clinician] to the Cloz clinic, are relatively stable. If they’re not, then they’ve usually been picked up or something has happened. So the focus can be off the mental health symptoms just a little bit.* (Focus Group 1, male participant 4)
	Priority is addressing mental health.	*The risks take a priority, mental health risks.* (Focus Group 3, male participant 2)
**Beliefs about capabilities**	Addressing behaviours with consumers and/or achieving behaviour change is too difficult to achieve	*It’s harder for the client that’s put on the weight, that doesn’t feel great, has still got a lot of symptoms present; it’s harder to motivate those people.* (Focus Group 1, male participant 1)
**Beliefs about consequences**	Lack of clarity regarding if other clinicians/services will manage outcomes.	*From our perspective there’s no guarantee that even if you pass information onto the GP that it’s going to be addressed.* (Focus Group 3, male participant 1)
**Memory, attention and decision processes**	Difficulty determining when providing preventive care is appropriate; considering consumer acuity, mental state, and rapport.	*I think it’s more - [properly] that when then their mental state is getting a stable stage then you can discuss…* (Focus Group 3, female participant 3)
	Too many tasks/competing tasks affecting ability to provide routine preventive care.	*I think it’s because of the workload of people in acute teams. Our number one thing is risk, mental health risk… around suicide risk…Yeah.…vulnerability… harm to others. I don’t think we really consider as close to the forefront of our mind the long-term risks of unhealthy lifestyles.* (Focus Group 3, female participant 1)
**Environmental context and resources**	Inadequate computer-based systems for recording and monitoring preventive care provision.	*(F2) So even though that I’m taking that information for the metabolic…[M3] it’s not going anywhere because I’m not monitoring - there’s no… charting attached to it.”* (Focus Group 3, male participant 3 and female participant 2)
	Insufficient time in mental health consultations.	*That’s why I think what [Male 1] and I were saying, alluding to, is that you don’t want to do it because then all of a sudden… you’re spending five days trying to work out their dietary needs and you’ve got 10 other clients’ mental health state deteriorating… because you’re consuming all your time on…* (Focus Group 3, male participant 3)
	Insufficient training, including induction training, on policies/guidelines/systems for provision of preventive care.	*Yeah, yeah, the disorientation – that’s a Freudian slip [laughs]. The orientation, that wasn’t involved in, so you found out as you went.* (Focus Group 2, female participant 1)
**Social influences**	Belief consumers would not be receptive to preventive care/beliefs about how consumers will react or respond.	You do get the occasional person who will go what the **** has this got to do with my mental health. (Focus Group 2, female participant 3)
**Emotion**	Feelings of fear and stress from completion of forms.	*It’s actually soul destroying… To be quite truthful… If you really sort of dwell on it for too long.”* (Focus Group 2, female participant 3)
**Optimism**	Despite own ability, expect factors will prevent routine preventive care to consumers.	*When they do become stable and settled and that’s what I’m saying, that’s when we actually… looking to discharge them.* (Focus Group 3, female participant 2)
**Reinforcement**	Lack of service level consequences/implications for not providing preventive care for consumers	*I suppose if someone said you either work and get paid or you can, you know, not work for us… I might force myself to do it.* (Focus Group 2, female participant 3)
**Intentions**	Not having clear intentions to provide routine preventive care to consumers in the future	*You know, as appropriate. I’m not saying that I would walk in that… …that being the first thing on my mind. No. But, you know, it probably… With every person would come up.* (Focus Group 2, female participant 3)
**Goals**	Provision of preventive care (for SNAP risk behaviours) not a goal in routine consultations with consumers	*I think we’re probably all aware of the national standards for the physical health of mental health consumers in the community. But I certainly don’t think it’s prioritised…* (Focus Group 3, female participant 1)

***Note.*** Barriers mapped to 13 of the 14 domains (no barriers mapped to ‘behavioural regulation’)

Current electronic systems and tools were highlighted as inadequate due to being inflexibly scripted and not allowing for ongoing monitoring of health and improvements (*environmental context and resources*). Inadequate time both during consultations and in terms of individual workloads was frequently reported as a constraint (*environmental context and resources*). Clinicians reported receiving limited training in procedures, referral services and skills to provide preventive care and navigate electronic forms (*environmental context and resources*). This was reflected in reports of low awareness of how to use electronic tools, available referral services to connect consumers to and how to organise referrals (*knowledge*); as well as low confidence in providing preventive care (*beliefs about capabilities*). Clinicians indicated perceived lack of competence in providing ‘advice’ and ‘referral’, with clinicians not assessing for health risks as they felt they do not have the skills to provide further care if they identified risks (*skills*).

Clinicians reported concerns about continuity of care, and expressed they were uncertain that referral services could effectively manage and address consumer outcomes (*beliefs about consequences*). Barriers relating to consumers included the belief that some may not want to receive preventive care (*social influences*). At the service-level, it was perceived there was a lack of reinforcement to encourage provision of preventive care or consequences for not doing so (*reinforcement*).

### Strategies to increase preventive care provision (individual interviews)

**Fig. 1:**
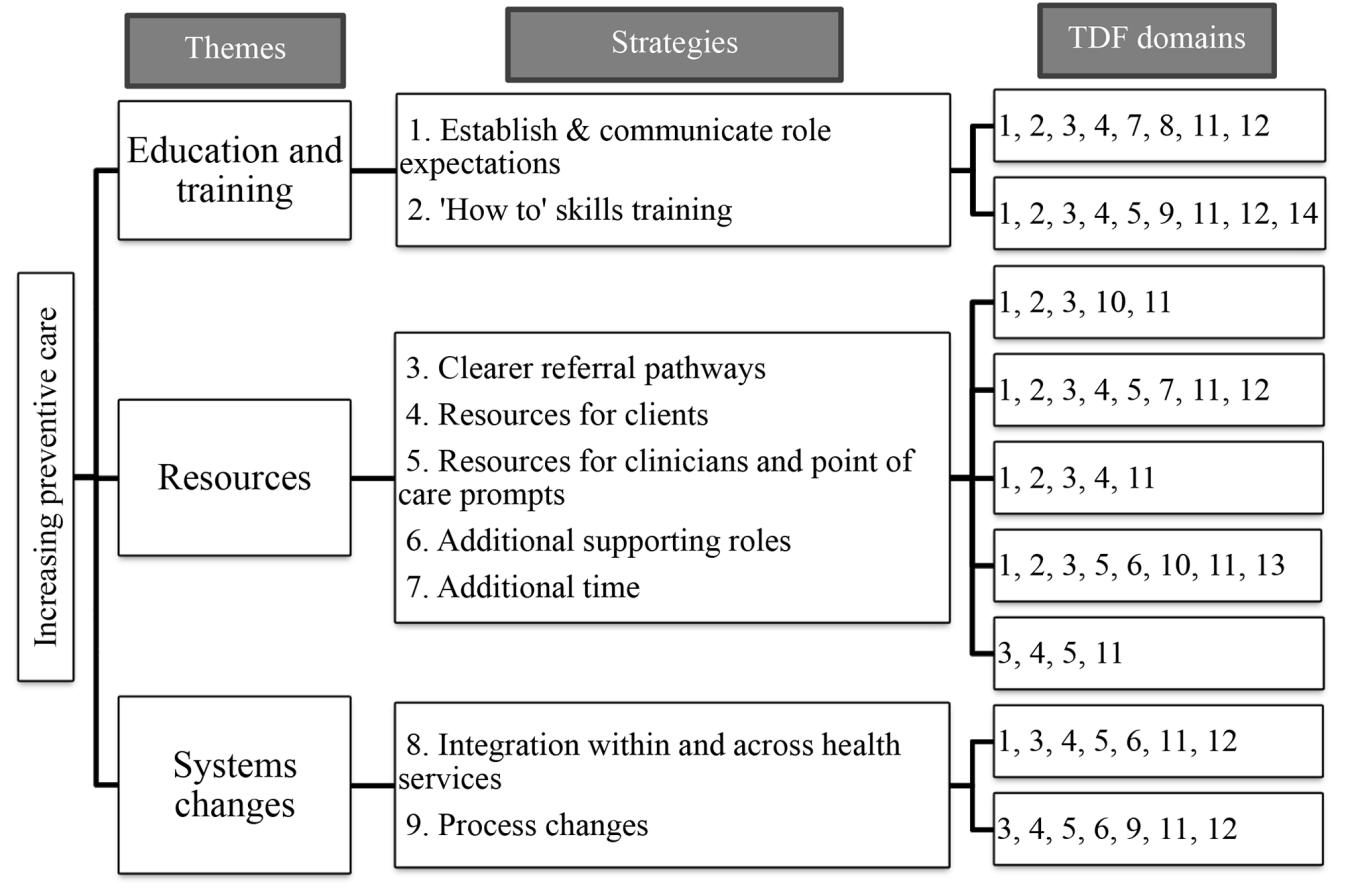
Clinician-generated strategies *Note*. Knowledge (1); Skills (2); Social/Professional Role and Identity (3); Beliefs about Capabilities (4); Optimism (5); Beliefs about Consequences (6); Reinforcement (7); Intentions (8); Goals (9); Memory, Attention and Decision Processes (10); Environmental Context and Resources (11); Social Influences (12); Emotion (13); Behavioural Regulation (14)

Clinician-generated strategies clustered in three themes, incorporating nine strategies that mapped to all 14 TDF domains (Fig. 1).

### Theme 1: education and training

#### Strategy 1: establishing and regularly communicating the expectation that clinicians have an important role to provide preventive care

Clinicians raised the need for further training to communicate preventive care as a key part of their role, including mandatory training reinforcing the links between physical and mental health. Other opportunities suggested were annual training and informal discussions in team meetings.

“*Making some kind of training package that is called ‘business’, making it part of mandatory training rather than just an optional extra. And really linking everything from that physical perspective or general health back to mental health.”*

(Participant 9, management position)

For new clinicians, a suggestion was to provide an orientation to the service that outlined physical health as a part of the role:

*“Have people that are new to our service to have at least a five-day period where they are clinically orientated to their role… and having people come in and out with areas of expertise, to have the dietician come in to talk about the importance of the better health check* [health risk assessment tool] *and the importance of working with the client around their physical healthcare, as well as their mental.”*

(Participant 8, management position)

#### Strategy 2: Providing skills training in how preventive care may be provided

The need to build confidence and competency specifically around ‘how’ clinicians can promote physical health was raised. Clinicians expressed the need to feel more ‘expert’ and skilled to talk to consumers about the impacts of health risks and how to make positive changes. Considerable discussion focused on the appropriate timing of when to provide preventive care.

*“They* [the clinician] *just identified the issue and then it’s, “Okay. Well, how do we get these fixed? Or how do we get this addressed?“ And I guess also back to education about what are the things that we’re looking out for? What are the priority areas, and how to ask the questions about it…*.*”*

(Participant 5, nurse)

*“Maybe if I got a bit more information and knowledge and expert… Someone telling me and how I could suggest to… clients to meet that.”*

(Participant 11, social worker)

Clinicians suggested more training on the available referral services and how to refer to them:

“*Information… About Get Healthy, information on coaching services, the referral like services. Quitline. Yep… And what actually happens to somebody and education surrounding that so that I can provide my clients with information*.*”*

(Participant 11, social worker)

### Theme 2: resources

#### Strategy 3: Clearer referral pathways

Clinicians spoke about the need for greater availability of referral services and clearer referral pathways. Ideally these services would be free to access, as this was viewed by clinicians as a consumer-level barrier.

*“Our community health teams need to be equipped with people that can help* [people with a mental health condition]. *We need to be able to refer our clients*.*”*

(Participant 13, psychologist)

#### Strategy 4: Resources and education for consumers to support behaviour change and establish expectations that mental health care includes consideration of health risk behaviours

Including workshops as part of service delivery was suggested, such as healthy cooking classes, physical activity sessions, and grocery shopping. Group activities were also noted to have additional benefits such as social interaction, supporting positive mental wellbeing.

*“Choosing 8 to 10 patients at a time and encouraging them to come… to a local community centre and do a six-week focus on cooking fresh food, and helping them steam veggies and helping them cook a piece of salmon or an eye-fillet steak… Whatever, so boil the* [potatoes], *or, “This is what you do to make mashed potato, it’s very very simple”…. Of course. That social interaction is extremely important… And being able to bounce things off each other.”*

(Participant 2, peer worker)

Educational resources (e.g., information brochures and simple healthy recipe cards) and practical tools (e.g., Nicotine Replacement Therapy) were recommended so clinicians have information and tools readily available.

*“Yeah, and even maybe like if there’s little sheets that I could provide to the clients, that has all of that in quite simple terms or some recipes or whatever of how you could incorporate some veggies into some meals, just simple kind of recipes, I guess, because a lot of people don’t wanna do elaborate cooking*.*”*

(Participant 11, social worker)

*… “but I think what’s really important if we’re gonna try and support people to reduce or quit smoking is to be able to give them methods to do that, so things like nicotine replacement therapy… to then give them a tool to be able to support them to do it would be really useful because otherwise cost-wise, it comes up as a really big barrier for a lot of our clients*.*”*

(Participant 15, dietitian)

Some clinicians suggested that new consumers could receive a service orientation, that includes setting the expectation that physical health will form part of mental health treatment. This may provide an opportunity to complete initial screening of health risks.

*“So I was thinking at the beginning of coming into the service maybe we could do that* [health risk screening]… *Yeah, that would make a lot of sense because if you’re asking them a lot of questions when they first come to the service, it would make sense to slot it in there.”*

(Participant 3, psychologist)

However, some did not support this idea, saying this should form part of regular practice and conversations with consumers. This was also evident for clinicians of the acute care team, noting that at commencement with the service some consumers may be too unwell to prioritise preventive care:

*“I think it sounds like a good idea. But I agree, I’m not sure what that would look like in the acute care team, because I think having it be a kind of priority… Yeah, they’re too unwell. I don’t know if they would be able to prioritize that education so early.”*

(Participant 6, social worker)

#### Strategy 5: Additional roles embedded in the service

Some clinicians supported the idea of a specific clinical role dedicated to providing preventive care to consumers:

*“I think it would be actually a brilliant idea if you have one designated role and that’s what that person’s role is to do, is to make sure that they have a half hour or an hour assessment, obviously depending when the person’s reasonably stable. To sit down and do that full assessment and then* [connect] *with… You could even incorporate that maybe some of the identified goals of that assessment could be incorporated into, Okay, well, here’s a group program. This group might help you with this and this one will help you with that. And we’ve got maybe the morning walk you could do everyday, or, you know… It has to be a targeted routine or planner for them.”*

(Participant 4, nurse)

However, there was concern about the feasibility (issues of funding, caseload, lack of existing rapport with consumers) as well as concerns that it may add further to fragmentation of care:

*“I think it could maybe be like passing the buck a little bit. And I think another thing that I hear from consumers is that they get very confused with how many people are involved in their care… So I would be mindful of that as well… I don’t see why we can’t focus on that stuff ourselves individually*.*”*

(Participant 6, social worker)

*“I’ve got some concerns around that* [the dedicated provider] *because I think we have so much awareness now about the link between physical health and mental health. So I honestly think it should be on everyone’s agenda and everyone should be talking about it.”*

(Participant 15, dietitian)

Other suggestions for additional support roles included embedded practitioners such as dieticians and exercise physiologists that consumers could be referred to for specialist support.

*“I’ve heard of other mental health teams… that have an exercise physiologist with their team. And I think that would be ideal… everyone can talk to clients about physical activity and give them guidance and options but I think in terms of having a dedicated person and dedicated time, having someone like that on board on our team would be amazing.”*

(Participant 15, dietitian)

The value of peer workers providing preventive care was also raised by one participant (a peer worker) who noted the benefits of being able to share their own lived experience:

*“When I explain to patients here that I’ve got lived experience, and I know what you’re going through, and I know how to help you because it’s happened to me in the past, they tend to drop their guard a lot.”*

(Participant 2, peer worker)

#### Strategy 6: Resources for clinicians and point of care prompts

Point-of-care prompts were suggested to assist clinicians when they are providing preventive care, such as a list of examples for how to incorporate exercise into daily activities, affordable and easy recipes, and referral services.

*“maybe just some examples about what we could talk about how to increase or some recipes… And you know, I guess those options for people to be able to have cheap vegetables or fruits that they can… Or more affordable options because finances is a very big barrier… So it really comes back down to or comes back to you having real clear knowledge of those all the things that are available…”*.

(Participant 11, social worker)

#### Strategy 7: Consideration of the time taken to provide preventive care in workload planning

Most clinicians reported that more time in consultations would allow for conversations about health behaviours. A dedicated consultation to discuss health behaviours was suggested:

*… “to have a dedicated session so that they don’t feel that pressure to discuss it, that it’s on the agenda all the time, but even to have a dedicated session where they know they’ve got the time to discuss these things… but I guess just some protected time around it to maybe have the conversations…*.*”*

(Participant 15, dietitian)

### Theme 3: systems changes

#### Strategy 8: Improved integration both within the mental health service and across the health care system

The need for preventive care conversations to be integrated with mental health treatment was noted, reinforcing the benefits of positive health behaviour change on mental health. One clinician suggested that reviews of preventive care being provided could be embedded in existing meetings where consumer progress is discussed.

*“if it were something that we were talking about each time we saw the patient and it was included in clinical review, an expectation at clinical review that we would discuss it with the team whether were making progress or not, and can other people make any suggestions, and so it becomes everybody business*.*”*

(Participant 12, nurse)

However, it was noted that embedding preventive care into routine practice needs appropriate organisational support and funding, including guidance and funding/resources from government organisations that oversee service delivery.

*“If services go and introduce little things ad hoc… so, it’s something that could be acknowledged in the either existing documentation suite, or endorsed by Ministry of Health, would be useful for people in my position who were trying to implement policy and making sure that we’re adhering to stuff. There’s enough ministry requirements, let alone a local initiative addressing something extra, I’d be really keen to have it included in existing frameworks or, and making it easy*.*”*

(Participant 9, management position)

*“If the powers that be were really serious about this, they would put in the resources. It’s a serious issue.”*

(Participant 10, nurse)

The issue of care fragmentation was raised: a lack of coordination across the different services involved in a person’s treatment. Clinicians noted the need to improve coordination and communication between services e.g., GPs, CMOs and referral services; however, no specific ideas for strategies to address this were shared by participants.

*“I think there’s a big role like a family or the other care involved should be all linked together.”*

(Participant 1, nurse)

#### Strategy 9: Improvements to streamline processes of care for consumers and clinicians

Despite there being large variability in consumers using the service, assessment tools were reported to take a ‘one size fits all’ approach. The following were suggested to improve existing tools: tick boxes to acknowledge if the assessment is not appropriate (e.g., due to immediate mental health risks), a method for monitoring changes/improvements over time, and streamlining existing forms to incorporate health risk assessment as part of initial intake assessment. An ‘alert’ was suggested to remind clinicians when health risk assessments were due.

*“making the better health tool* [electronic tool] *meaningful and useful and actually providing some of the information and referral information that is actually needed*.*”*

(Participant 14, management position)

“…*but if there can be some sort of alert system that comes up on them…*.*”*

(Participant 11, social worker)

To aid consumers in navigating the complexity of the health care system to access preventive care, a suggestion was to have a dedicated clinician or service clients can speak to about health risk behaviours.

*“Again, it’s having someone who they can identify with, they know. [name] is the girl to see about the NRT. When I last* [tried], *I made an appointment to see [name], and [name] got me what I wanted, and I was able to have another crack, but if we make things easier for people…*.*”*

(Participant 10, nurse)

## Discussion

This is the first qualitative study investigating community mental health staff suggested strategies for increasing provision of preventive care specifically for key chronic disease risk behaviours: smoking, poor nutrition, harmful alcohol use, and physical inactivity. The novel use of the TDF in this specific context enabled comprehensive identification of factors influencing care provision. Several barriers were identified, with the greatest number being within domains of *knowledge* and *environmental context and resources*. Strategies suggested aligned with themes of training, resources and systems changes; mapping to all 14 TDF domains. Findings may provide guidance for future research and quality improvement projects seeking to increase implementation of preventive care; and highlight the need for greater recognition and resourcing as a priority within mental health treatment.

Clinician views of key barriers to preventive care provision were largely consistent with previous quantitative and qualitative research that has focused on physical health more broadly [12, 14–18]; most notably including perceived low confidence and skills, particularly for steps beyond assessment of behaviours (i.e., ‘advice’ and ‘referral’), as well as a limited knowledge of available referral services. Consistent with previous research [8, 40, 41], competing priorities was raised. This study adds new knowledge regarding the varied perspectives of clinicians based on their role and/or the consumers they typically see. Participants from the acute care team raised the issue of competing priorities and concerns regarding timing of when to provide preventive care.

This study and previous research [8] support the need for clinician training to implement preventive care as part of routine practice. Two key areas for training were recommended by participants: [1] building clinician skills and capacity and [2] setting expectations that preventive care is an integral component of mental health care. Given the difference in perspectives across clinical roles, training could be tailored to consider clinicians’ specific role and clientele. Clinicians perceived greater resources were needed at the service/health system-level (e.g., clearer referral pathways and more staff), clinician-level (e.g., more time in consultations and point of care prompts) and consumer-level (e.g., educational brochures and group workshops). While the present study only included one peer worker, they noted the benefits of sharing their lived experience to guide preventive care delivery. Peer workers have an increasingly important role in providing mental health treatment and available research suggests that peer-delivered interventions are effective in promoting lifestyle change for this population group [42, 43]. Further research to explore the perspectives of peer workers regarding their role and capacity in providing preventive care is warranted.

The potential of embedding a dedicated preventive care provider in the service was also raised, though there were mixed views regarding this approach. Previous research has found that integrating a dedicated preventive care provider in a community mental health service resulted in significantly increased provision of preventive care [30]. However, some staff in the present study noted limitations of this approach, such as perpetuating the lack of integration between mental and physical health care (by continuing to separate the roles).

The need for greater integration both within mental health service delivery and with external services was raised by participants, aligning with previous studies [16, 44] and positional statements [45] that indicate a need for broader health system reform. The idea of a coordinated or shared approach, where health professionals across service settings work collaboratively to provide care, has been recognised as a national priority [46, 47], as well as by consumers [48] and expert stakeholder [45] groups.

The findings clearly highlight the need for further investment in resources, skills development and improved coordination of care to provide practical and tangible support for preventive care provision, as well as to strongly communicate it as a priority. While guidelines and policies exist exhorting the provision of preventive care within mental health services [3], a consistent finding of research is that while guidelines and policy are important, they need to be augmented with implementation support strategies to achieve change in clinical practice [49–51] [52]. There is a need for governing authorities and health services at a state and national level to examine the possible models of preventive care provision, and the available evidence for their effectiveness and cost-effectiveness. Investment and advocacy into further research that includes co-design with end-users is crucial to inform decision-making.

Limitations include that at the time of the focus groups in service one, a randomised controlled trial had recently been conducted in that service to evaluate the effectiveness of embedding a dedicated preventive care provider in the service [30] and may have impacted clinician views of this as a potential strategy. Transferability should be considered in this context; and may also be impacted by other contextual factors such as the presence of a preventive care policy and the available electronic tools. Information was not collected regarding the team each participant worked in (acute, community or rehabilitation), however, where this was raised by participants during discussions this was included in the results demonstrate where views may have been different. The study is strengthened by its inclusion of clinicians from varied roles and professional backgrounds, as well as the mixed data collection methods that enabled exploration of both individual and group views [53]. The sample had sufficient information power, supported by the use of a theory (TDF) to guide analysis, high specificity of experiences among the sample, and high-quality dialogue (driven by having a qualitative expert facilitate, and content expert co-facilitate the focus groups) [54].

## Conclusions

Mental health clinicians identified a range of strategies they felt would address barriers and support the provision of preventive care. Successful implementation of preventive care will require it to be integrated as a priority and key component of mental health treatment. Development of initiatives to increase its provision could be guided by the perspectives in this study, as well as that of the greater evidence-base regarding effective implementation strategies to increase preventive care provision and support change in clinical practice more generally [55]. Future research is also needed to explore the views of mental health consumers regarding such initiatives.

### Electronic supplementary material

Below is the link to the electronic supplementary material.

Supplementary Material 1

## Data Availability

The datasets supporting this article are available upon reasonable request to the corresponding author (CF).

## List of Abbreviations

TDF: Theoretical Domains Framework
AAR: Assess, advise, refer
SNAP: Smoking, poor nutrition, harmful alcohol consumption, physical inactivity
NDIS: National Disability Insurance Scheme
