# Supplementary material for: Increasing chronic disease preventive care in community mental health services: clinician-generated strategies
Source: BMC Psychiatry. 2023 Dec 11;23:933. doi: 10.1186/s12888-023-05311-9 (PMC10714530; doi:10.1186/s12888-023-05311-9)
Supplement: Supplementary file 1 — Supplementary Material 1 [file 12888_2023_5311_MOESM1_ESM.docx]

**Online Supplementary Material**

**Additional File 1. Focus group discussion guide**

# Introduction

*Introduction to study and research team. This should include a clarification of our roles including our interface and relationship with the health services to ensure that participants feel completely free to talk about their organisational climate, colleagues, discrepancy between expected and actual performance etc.*

- As you know, our team is interested in the physical health of people with a mental illness.
- The life expectancy of people with a mental illness is much shorter than the general population, and this has a lot to do with key risk behaviours such as tobacco smoking, poor nutrition, risky alcohol consumption and physical inactivity
- We’d like to hear from you about the ways that you think mental health services could have a role in addressing the risk behaviours of your clients
- What we would appreciate talking to you about today is what things may help you and the service address these risk behaviours with your clients, as well as the things that make it difficult to do so
- A better understanding of these factors could help inform the development of tools or interventions to assist your service in providing the best possible, holistic care to your clients.

*General informed consent process (consent form and demographic questionnaire completion).*

- This is an open forum where you are completely free to talk about your experiences and opinions.
- We want to know what is not working, so it would be great if you open up and talk about things that are going on/your experiences
- The more honest you are, the more we can learn from your experiences and tailor future research investigating interventions/supports to suit your needs.
- We won’t be feeding back any identified information to the mental health service.
- Participation is voluntary, you may withdraw at any time
- We will be recording the audio - all information collected will be non-identifiable
- Complete demographics questionnaire

*Specific introduction to focus group content and format, and “rules”.*

- Including a mention of: what gets said here, stays here – respect the views expressed by your colleagues today. Do not share what others have said when you leave.

*State research aims.*

- The discussion today will be around the role that the mental health service could have in helping identify and address these risk behaviours systematically and routinely for all its clients
  - We want to hear from you about how this kind of care could be provided to your clients, including what may help you do so and what things make it hard to do
  - We are interested in hearing about these barriers and facilitators both for yourselves individually, but also at a wider service level
- This kind of care could include things like:
  - Assess
  - Advice
  - Referral – Get Healthy and Quitline
- The risk behaviours we are talking about are: tobacco smoking, inadequate nutrition, harmful alcohol consumption and physical inactivity.

# Key Topics

## First, I would like to hear from you as to how do you, as clinicians, perceive your role and the role of the mental health service in providing routine and systematic care for the health risk behaviours (HRBs) of your clients

- How well does providing such care fit in with your role?
- Tell me a bit about how you, in a given consultation, decide whether to address HRBs?
- Tell me how addressing HRbs fit on to your list of goals or priorities for client consultations.
- Does providing such care carry any negative / positive connotations for you?
- At a service level we could consider – what initiatives or programs does the mental health service have in place?

## I would now like to hear from you as to what the perceived barriers are to providing such care

*(open-ended – prompt from list after initial discussion)*

- Lack of knowledge/familiarity of how care should be provided/what kind of care/what it should ‘look like’
- Lack of knowledge/familiarity of the criteria for risk across the 4 HRBs
- Lack of skills/training in providing such care (consultation, assessment, MI)

⭢ lacking confidence

- Not perceiving to make an impact on clients’ lives

⭢ lacking confidence in the ability to bring about change

- Difficulties due to impact of: colleagues, management, workplace cultures
- Lack of resources
- Organisational policies

# Closure

Closing summary/Ask for additional comments

**Additional File 2. Pre-reading material for individual interviews**

**

**Additional File 3. Individual interview discussion guide**

## 1. Introduction

- As you know, our team is interested in the physical health of people with a mental illness
- The life expectancy of people with a mental illness is much shorter than the general population, and this has a lot to do with key risk behaviours such as tobacco smoking, poor nutrition, risky alcohol consumption and physical inactivity
- We recently conducted some focus groups, to explore staff views on what may help you and the service to address these risk behaviours with your clients, as well as what makes it difficult to do so
- In these groups we learnt that staff feel that the physical health of their clients is extremely important, and agree that the service has a role in helping clients to engage in positive health and lifestyle behaviours. However, we heard from the staff that there are a number of barriers, or factors that can make it difficult to do so.
- We now want to talk to staff in more depth about what can be done to make this care ‘happen’ systematically and routinely for clients of this mental health service

## 2. Opportunity to discuss own barriers

- Firstly, I would like to just give you a brief summary of the type of barriers to care that were talked about in the focus groups
- A few examples of barriers that staff suggested in these focus groups included:
  - Competing priorities when addressing both mental and physical health all in one session
  - Not feeling confident in providing this care (I’m not the expert) – e.g. not knowing what to say when advising someone to change their health behaviours
  - Not knowing where you can refer your clients onto for some ongoing support for their health and lifestyle behaviours, OR not feeling confident in the ability of these services to support clients
  - Not being sure how the electronic tools in CHIME (e.g. better health tool) work – e.g. not knowing how to send a referral to the Quitline or Get Healthy
- Do these type of barriers resonate with you in your role?
- Are there any additional things that you feel prevent you, or the mental health service, provide care to address health risk behaviours? (these may be factors specific to you and your role, or maybe factors around your team, or the organisation as a whole)

## 3. ‘Unstructured’ discussion of potential solutions

- Now that I’m starting to develop a picture of what type of things stop you from providing this care to an extent that you perhaps would like to , I’d like to spend the remainder of this interview talking about some of the ways we could make this happen
- These solutions or ideas you may have may not necessarily relate just to the barriers we’ve already spoken about already
- Ideal world scenario – to shift thinking from barriers to solutions (“I would like to start this talk about solutions by asking you a question: *“If I gave you a magic wand, what would you change to make it possible for you (or staff here in general) to comprehensively address physical health risk behaviours in a routine manner, so in terms of both assessment, advice and referral?”*

## 4. ‘Structured’ discussion of potential solutions

- Looking at this diagram (pre-reading), what would be the most important level at which changes could be made? What would be the 2^nd^?
- So talking about this [level mentioned by participant], tell me about some of the things you feel could be put in place.
- Prompt across the visual prompt ‘levels’
  - I’ve noticed you’ve generated some really good solutions which come under x level, can you think of anything at x level?
  - Is there anything else you can think of at x level?
- Out of all the solutions you have spoken about today, what would be your top solution? Or top 3?
  - Now, how would you relte the do-ability of this for your service, on a 1-5 scale?
